# Prototype of Data Collector from Textronic Sensors

**DOI:** 10.3390/s23249813

**Published:** 2023-12-14

**Authors:** Ewa Korzeniewska, Rafał Zawiślak, Szymon Przybył, Piotr Sarna, Anna Bilska, Mariusz Mączka

**Affiliations:** 1 Institute of Electrical Engineering Systems, Lodz University of Technology, Stefanowskiego 18 Street, 90-537 Lodz, Poland; 2 Institute of Automatic Control, Lodz University of Technology, Stefanowskiego 18 Street, 90-537 Lodz, Poland; rafal.zawislak@p.lodz.pl; 3 Faculty of Electrical Electronic Computer and Control Engineering, Lodz University of Technology, Stefanowskiego 18 Street, 90-537 Lodz, Poland; szymprzy@outlook.com (S.P.); sarna2000piotr@gmail.com (P.S.); anna.emma.bilska@gmail.com (A.B.); 4 Department of Electronics Fundamentals, Faculty of Electrical and Computer Engineering, Rzeszow University of Technology, 35-959 Rzeszow, Poland; mmaczka@edu.prz.pl

**Keywords:** textronics, wearable electronics, data collectors, impedance sensor

## Abstract

In the era of miniaturization of electronic equipment and the need to connect sensors with textile materials, including clothing, the processing of signals received from the implemented sensors becomes an important issue. Information obtained by measuring the electrical properties of the sensors must be sent, processed, and visualized. For this purpose, the authors of this article have developed a prototype of a data collector obtained from textronic sensors created on composite textile substrates. The device operates in a system consisting of an electronic module based on the nRF52 platform, which supports wireless communication with sensors using Bluetooth technology and transmits the obtained data to a database hosted on the Microsoft Azure platform. A mobile application based on React Native technology was created to control the data stream. The application enables automatic connection to the selected collector, data download and their presentation in the form of selected charts. Initial verification tests of the system showed the correctness and reliability of its operation, and the presented graphs created from the obtained data indicate the usefulness of the device in applications where measurements and recording of impedance, resistance, and temperature are necessary. The presented prototype of a data collector can be used for resistance, impedance, and temperature measurements in the case of textronic structures but also in other wearable electronic systems.

## 1. Introduction

Sensors and wearable electronics are terms that refer to small electronic devices that can be worn on the body or embedded in clothing, jewelry, watches, wristbands, glasses, and other accessories. With these, various physiological, environmental, or behavioral parameters of the wearer, such as heart rate, blood pressure, body temperature, blood glucose levels, body position, movements, physical activity, or location can be monitored [1,2,3,4,5,6,7,8]. Communication between them and other electronic devices is possible using wireless technologies such as Bluetooth, Wi-Fi (Wireless Fidelity), NFC (Near Field Communication), or RFID (Radio Frequency Identification) [9].

The sensor and wearable electronics market is one of the fastest-growing segments of the consumer electronics market. According to some reports [10,11,12], the size of the wearable sensor market is estimated at USD 3.88 billion in 2023 and is expected to reach USD 6.97 billion by 2028, with a Compound Annual Growth Rate (CAGR) of 12.41% over the period to 2030. Some of the key growth drivers for this market are the increasing demand for smaller, smarter, and cheaper sensors, growing awareness of health and wellness, advances in sensor technology and battery performance, and the emergence of new applications such as remote patient monitoring and infant care [13].

In the case of small wearable devices equipped with both power supply and signal transmitters and receivers, there is no need to transmit data over further distances, as the data processing takes place within the devices. The situation is different for textronic systems, where there is a need to transmit information on measured quantities to receivers outside the measurement system, where the data will then be received, processed, and analyzed without the need to maintain a wired network connection.

Wearable electronic elements embedded in textiles are integrated into textile materials or applied to their surface by various techniques such as weaving [13], embroidering [14], sputtering [15], printing [16], or thin film deposition by spin-coating [17], chemical vacuum deposition [18], or physical vacuum deposition [19]. Among other things, these components can act as sensors and can communicate with other electronic devices via wireless technologies.

Wearable sensors can monitor various physiological or environmental parameters of the user, such as heart rate, blood pressure, body temperature, blood glucose levels, body position, movements, physical activity, or location. Among the group of sensors are impedance sensors, which use changes in electrical impedance (resistance and reactance) in human tissues to measure various physiological parameters such as heart rate, blood pressure, hydration levels, stress levels, sleep quality, or detection of developing pathogens [2,20,21,22].

The data from wearable impedance sensors are transmitted to mobile devices using radio signals of a specific frequency and power. These signals are modulated and encoded by the sensor transmitter, then received and decoded by the mobile device receiver. Depending on the wireless technology used for communication, data can be transmitted in different modes (e.g., asynchronous, synchronous, half-duplex, full-duplex) and at different transmission speeds (e.g., from kilobits to megabits per second).

The data collectors are used for gathering and storing data from various sources, including wearable sensors. They can work in different ways depending on the type and purpose of the data collection. Some wearable sensors can connect to a smartphone or a computer via Bluetooth and transmit the data they collect to an app or software. The app or the software can then process, analyze, and display the data for the user or send it to a cloud server for further analysis. For example, the STEVAL-WESU2 wearable sensor platform from STMicroelectronics N.V. (Geneva, Switzerland) can connect to a smartphone app via Bluetooth and send data such as motion, pressure, and temperature [23]. Another possibility of communication of wearable sensors is using a wireless network such as Wi-Fi or cellular and sending the collected data directly to a cloud server or a web service. The cloud server or the web service can then process, analyze, and store the data for the user or share it with other authorized parties. For example, Google’s Project Jacquard [24] uses conductive yarns and miniature electronics to create smart fabrics that can connect to a smartphone or a cloud service via Wi-Fi and enable gesture recognition, contextual awareness, and haptic feedback [25]. Data collectors can also work with different types of data, such as numerical, textual, audio, video, or image data, and use different methods to process and analyze the data they collect, such as artificial intelligence (AI), machine learning (ML), deep learning (DL), neural networks (NN), or sensor fusion algorithms [23,25].

The research examples presented above confirmed the possibility of creating an impedance textronic sensor, sensitive to changes in resistance and capacitance as a function of frequency, for use, for example, in the detection of *Staphylococcus aureus* [2]. To use such a sensor, it was necessary to create an entire electronic system that would measure these parameters and send them to a dedicated mobile application that would visualize the measurement values. There are no solutions on the market that could be implemented in the designed sensor using interdigital electrodes produced on flexible textile substrates. The proposed solution is innovative and can be used in wearable electronics to convert information about changes in electrical parameters into visual information.

The purpose of this paper is to describe a prototype of a data collector designed to measure the impedance values of textronic sensors made with physical vacuum deposition technology such as that described in the paper [2], then transfer the data to a database using Bluetooth technology. To analyze the transmitted information, a mobile application was created using React Native technology. There are no such solutions available on the commercial market.

## 2. Concept and Assumptions of the Project

The idea of the project was to develop and implement a system capable of acquiring data from sensors to obtain information on changes in selected measurement parameters such as impedance or temperature. Sensors installed on textronic structures were to communicate with an electronic module that would convert the obtained electrical signals into specific measurement values of selected parameters. The acquisition process was to be controlled using a mobile application and the obtained data were collected and processed in a database available from a pre-defined user level.

**Figure 1:**
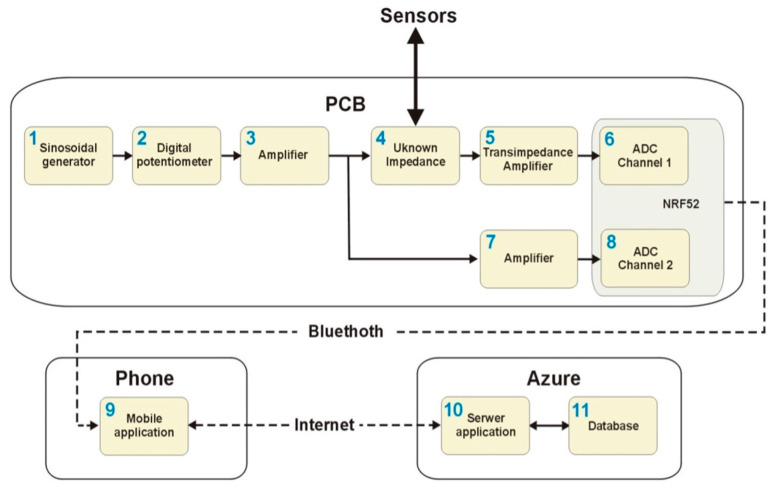
Block diagram of the system.

Based on the above concept, the technical descriptions available in the literature, and our own experience gained during laboratory measurements of textronic structures, a system was developed. The block diagram of the system is illustrated in Figure 1.

To measure the impedance of the interdigital sensors (see block 4), a suitable measurement system needed to be created. The auto-balancing bridge technique was used to provide the required parameters, which was designed based on the Texas Instrument Design Guide: TIDA-060029 LCR Meter Analog Front-End Reference Design [26]. The auto-balancing bridge was chosen due to good accuracy in a wide range of impedances and frequencies [26]. To create an auto-balancing bridge, a first block diagram (PCB) was created to divide the hardware into smaller functional parts. 

To save and store the measurement data from the electronic system, it was necessary to create a database. It was performed with Microsoft Azure, enabling quick configuration and tools to simply create a database structure. However, to communicate the textronic device with the above software, it was necessary to create a mobile application that would also allow for a clear presentation of the measurement results. React Native software (version 0.70.6) was used to create a program for the smartphone, which communicates with the Microsoft Azure database using the NestJS framework. Through this connection, the application retrieves data from the measuring system, then prepares and adds it to the individual table in the database.

## 3. Hardware Implementation

**Figure 2:**
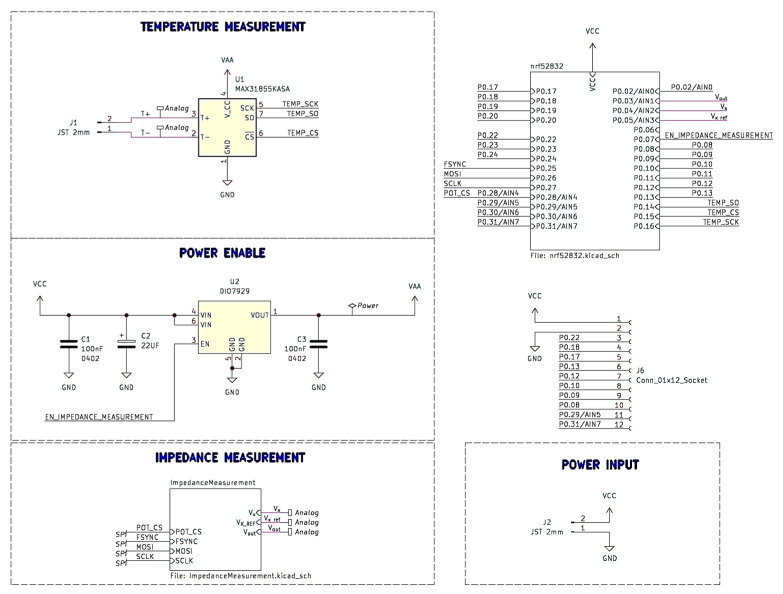
Electrical and conceptual diagrams of the basic functional blocks planned for the PCB.

The main component of the PCB is the nRF52832 SoC (System-On-Chip) (see Figure 1), which is responsible for wireless communication, measurement, and processing of analog signals. The functional diagram of this chip and the electrical and conceptual diagrams of the temperature and impedance measurement modules are shown in Figure 2. The same figure also contains illustrations showing the control of the power input and output modules planned within the system. For this purpose, the DIO7929 system was used, which operates in the high voltage side switching system used to turn on the power supply to the measurement circuits.

A MAX31855KASA integrated circuit, which is characterized by low energy consumption and the ability to support a wide range of thermocouples, was used to measure the temperature. 

**Figure 3:**
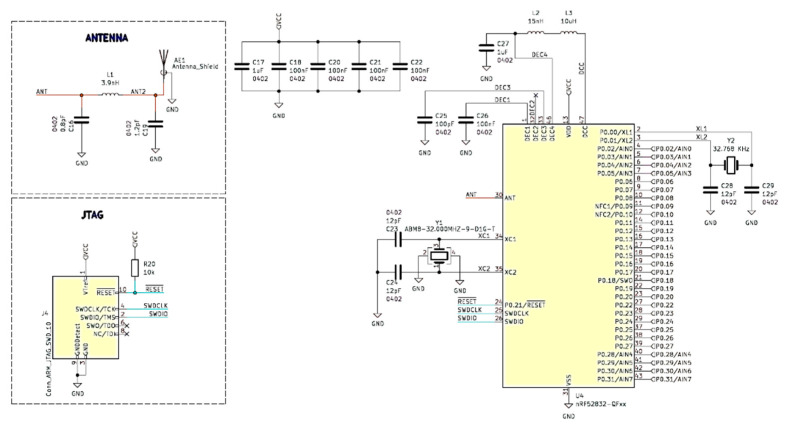
nRF52832 microcontroller and necessary components.

**Figure 4:**
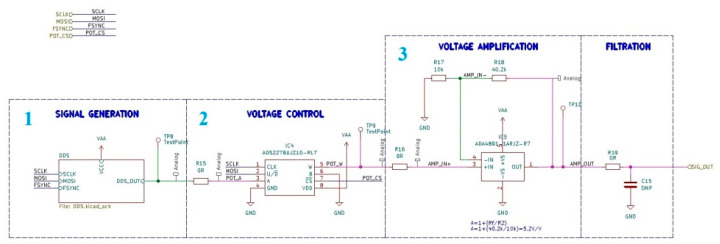
Electronic implementation of the blocks necessary to generate and control the sinusoidal voltage signal.

**Figure 5:**
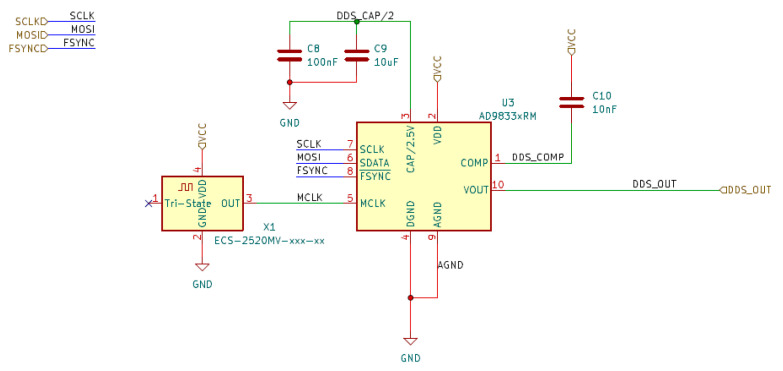
Schematic of programmable waveform generator based on the AD9833 used for sinusoidal voltage signal generation.

Based on the block diagram shown in Figure 1 and the capabilities of the nRF5282 IC, a system PCB was designed using the open-source software KiCad [27] (https://www.kicad.org, accessed on 14 August 2023). The results of the work in this area are shown in Figure 3, Figure 4 and Figure 5, which are illustrations of the hierarchical electrical schematic of a multilayer PCB. In the main sheet shown in Figure 3, a sub-sheet has been added, in which the nRF52 module is designed with the necessary components for proper operation. On the same sheet, an electrical diagram of the antenna module and JTAG (Joint Test Action Group) interface has been included.

As an antenna, the Texas SWRA117D 2.4 GHz PCB antenna [28] was used due to ease of implementation and reduction in cost due to requiring only a matching network to completely implement the antenna circuit.

The arrangement of blocks 1–3 (see Figure 1) is illustrated in Figure 4. The signal generator module is realized based on a specialized AD9833 integrated circuit. It is a programmable low-power sine wave signal generator capable of generating a signal from 0 MHz to 12.5 MHz with 28-bit frequency resolution and a programmable phase. A detailed electrical diagram of the sinusoidal signal generation module is shown in Figure 5.

The voltage generated by block 1 is controlled by a digital potentiometer AD5227BUJZ10, which allows control of the signal amplitude with a resolution of 6 bits using a unidirectional SPI interface.

Then to ensure low output impedance before unknown impedance, the ADA4891 rail-to-rail operational amplifier is used. This operational amplifier was chosen based on its low input bias current, which is required for measurement of high impedance and high open-loop gain for ensuring stable operation [29]. An additional resistor (R16) in series was added to improve the stability of the circuit and the impedance measurement. The signal after this additional resistor is buffered using an operational amplifier (ADA4891). The signal in this node is measured by nRF52 to estimate its phase and amplitude.

**Figure 6:**
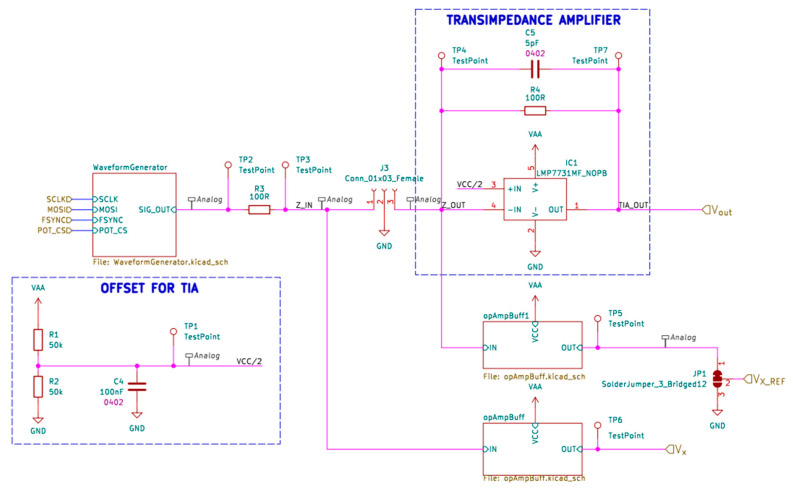
Schematic of the impedance measurement circuit.

The electronic implementation of blocks 3–7 (see Figure 1) is shown in Figure 6. After the signal passes through an unknown impedance, the current, flowing through it, is converted to the voltage signal using a transimpedance amplifier. The voltage signal is captured using the internal analog-to-digital converter of nRF52.

The excitation signal is passed through the known resistor (R3) and unknown impedance connected to the connector J3, then it will be passed to the transimpedance amplifier that converts current flowing through a measured impedance to a voltage signal (V_OUT_) and that signal is measured to calculate the unknown impedance. Additionally, the excitation signal before the measured impedance (V_X_) is measured. To calculate the impedance from those signals, it is necessary to estimate the phase and the amplitude of the signal.

**Figure 7:**
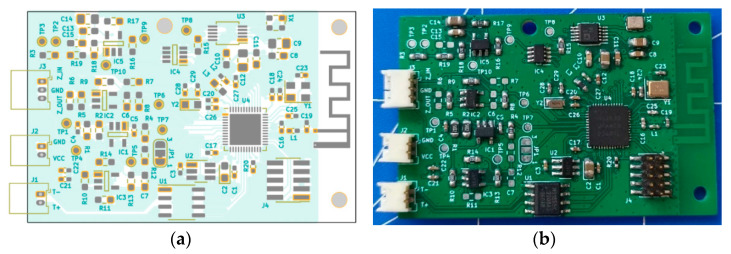
Final PCB design. (**a**) Board design used for impedance measurement and (**b**) assembled board.

The PCB seen in Figure 7 was designed on a four-layer board with all components on the top side. Connectors with 1.25 mm raster connectors were used and the overall dimension of the PCB was 49 mm × 31 mm.

All traces were designed to have 50 Ω impedance, and trace widths for given impedance were obtained from the PCB manufacturer website. On the right side of the PCB, an antenna and matching circuit was placed to separate the analog circuit and the antenna circuit as much as possible. All connectors are placed on the left side. 

## 4. Data Acquisition

Analog measurement is conducted using the internal ADC (analog–digital converter) of nRF52. A timer present in the nRF52832 triggers consecutive measurements on both channels, and oscillograms of the measured data are stored in the memory. After finishing the measurement of the full period of the excitation signal, the timer stops triggering measurements and the measurement is processed. 

The use of the internal ADC converter of the nRF52 ensured a reduction in project costs but did not reduce the measurement accuracy. The 12-bit Successive Approximation Analog-to-Digital Converter (SAADC) with a maximum sampling rate of 200 kHz, 8 configurable channels, and programmable gain provides sufficient accuracy for known applications in biomedical measurements [30,31].

In addition to the calibration process, particular attention has been given to power stability. Maintaining a stable power supply is paramount for accurate and consistent ADC measurements. Measures have been implemented to mitigate potential fluctuations in the power supply, thereby contributing to the overall precision of the system.

As the device will be in sleep mode at most times, the biggest impact on the power consumption is sleep current consumption [32]. In order to minimize current consumption in sleep mode, power to the measurement circuitry is turned off using DIO7929 high side switch, which allows the nRF52 chip to be the only chip that is left powered in sleep mode. Current consumption in sleep mode according to the online power profiler is 2µA [33], and DIO7929 current consumption when powered off should be <1 μA [34].

There are two modes of operation of this device. In the first mode, the device measures impedance at a fixed frequency and every data measurement is sent, enabling data updates of about 100 Hz depending on the excitation signal frequency. The second mode is the frequency sweep measurement, in which data are sent in bulk with every frequency sweep. The frequency at which the measurement is sent depends on the frequency sweep range and the number of measurements. The ADC needs to measure one period of signals in order to estimate the amplitude and phase of the signals, which takes an amount of time specified by equation t_{single measurement} = 1/f_{excitation}; then, this time is multiplied by the number of averaging functions used and by the number of different excitation frequencies that will be measured during sweep. Additionally, some delay is introduced between different excitation frequencies in order to give the sensor time to stabilize its response for a given frequency.

The amplitude is estimated, according to the manufacturer’s recommendations (Texas Instruments, LCR Meter Analog Front-End Reference Design, Dallas, TX, USA, 2019) [26], by multiplying the signal by a square wave with a value of unity with phases of 0° and 90° and calculating the average of the received signals. From these averages, the amplitude (using Equation (1)) and the phase (using Equation (2)) can be calculated [35]:

(1) $$\left|V\right|=\sqrt{V_{1}^{2}+V_{2}^{2}}$$

(2) $$\alpha =\tan^{-1}\left(\frac{V_{2}}{V_{1}}\right)$$

where:

|V|—amplitude of the signal

α—phase of the signal

V_1_—average value of the signal modulated by 0° unity magnitude square wave

V_2_—average value of the signal modulated by 90° unity magnitude square wave

From known amplitudes and phases of V_x_ and V_out_, impedance is calculated using Equation (3):

(3) $$Z_{x}=R_{REF}\frac{V_{x}}{V_{Out}}$$

where:

Z_x_—unknown impedance

R_REF_—value of the resistor used in feedback of the transimpedance amplifier and before measured impedance.

V_OUT_—complex voltage of the signal measured from transimpedance amplifier (measured at Channel 1, according to Figure 1)

V_X_—complex voltage of the signal measured before unknown impedance (measured at Channel 2, according to Figure 1)

After calculating the impedance system measures five times, the median value of those measurements is calculated. Then, from five of those medians, the average value is taken to get the final result.

### 4.1. Algorithm for Data Transmission

**Figure 8:**
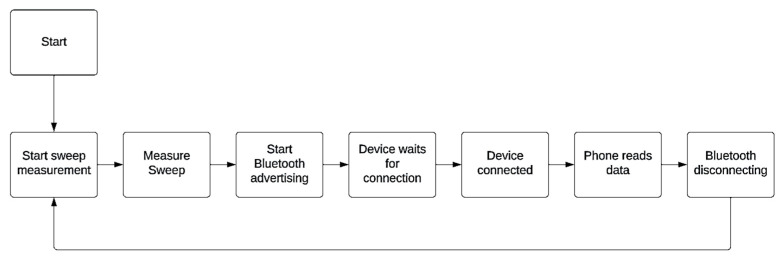
Flowchart of the microcontroller communication.

Communication between the device and the phone is created using Bluetooth. The flowchart of the microcontroller communication is presented in Figure 8.

The first device measures complete sweep data and, after finishing, starts Bluetooth advertising and waits for the phone to establish the connection; the phone then reads the data from the device and disconnects. After disconnecting, the devices start measurement again.

The mobile app collects the data from the textronic device to update the displayed information, such as graphs or numerical values on the home screen. It also sends new data in the appropriate format to the database, so the latest data can be seen if the connection to the device is lost and/or the device works offline.

### 4.2. Database

As part of the project, based on current sources [36,37,38,39,40], currently available database technologies were analyzed and compared. The effects of this work are illustrated in Table 1.

Based on the above research and analyses, it was concluded that the fully managed relational Azure SQL (Structured Query Language) database is the most suitable for our project. It is compatible with Microsoft SQL Server. Moreover, it offers scalability and high availability. The Azure platform includes a range of advanced security features, including role-based access control, network security groups, and protection against DDoS (Distributed Denial of Service) attacks. It also provides seamless integration with other services and Microsoft tools (e.g., Visual Studio). When selecting the most suitable database for the project, the following were considered: Azure SQL Database, MySQL, PostgreSQL, Microsoft SQL Server, and Oracle.

**Figure 9:**
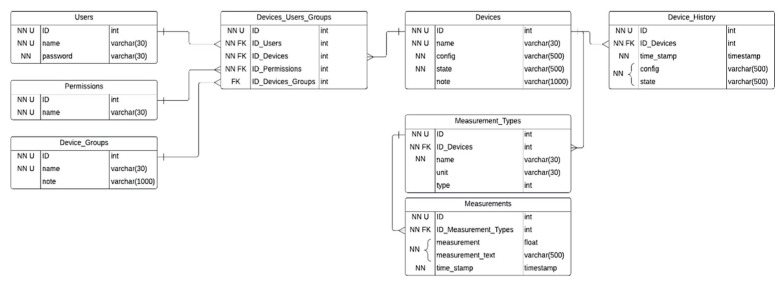
Block diagram of the database.

The technical structure of the database (see Figure 9) has been created for monitoring and analyzing the parameters of measuring devices. The technical structure consists of multiple tables.

The first step is to maintain security and access control. We used four tables to precisely describe the rights of the users. The first table is the ‘Users’ one. It stores the users’ data, including their ID (identification), name, and password. The second one is the ‘Permissions’ table. It defines the access levels in the system, such as ‘Admin’, ‘User’, and ‘Observer’. This allows different levels of access to be defined, which can be controlled by mapping users to the appropriate permissions in the ‘Permissions’ table, a group of devices from the ‘Device_Groups’ table, or even a single device from the ‘Devices’ table. The table ‘Devices_Users_Groups’ allows the precise definition of user capabilities regarding data and device operations. The ‘Device_Groups’ allows grouping of the devices. Each group can have a description that makes it easier to understand the purpose of the device category. The most important table is the ‘Devices’ table, in which monitoring devices are registered, storing their name, configuration, and current state. The device identifier ID is a key used as a foreign key in other tables. The technical structure also includes a table of ‘Device_History’. Devices can have different configurations, such as sampling frequency or other settings. The ‘Device_History’ section allows for storage of the configurations and states of devices which can be crucial for future analysis. The different measurement types such as temperature, resistance, or reactance are defined in the ‘Measurement_Types’ table. Each measurement type has an associated unit. In the ‘Measurements’ table, the numerical values measured by the devices are stored together with the measurement time. For some measurements, the values are stored in text form; for this, we use JSON (JavaScript Object Notation) formatted data.

This comprehensive database structure creates a solid foundation for monitoring, analyzing, and managing the parameters of measuring devices. It enables efficient data collection, access control, and trend analysis. It is a key tool for modern technical systems, which rely on accurate information and effective data management.

### 4.3. Mobile Application

The mobile application for remote control of the system was developed using the React Native environment. It is a JavaScript framework for writing real, natively rendering mobile apps for iOS (iDevice Operating System) and Android. It is based on React, which includes JavaScript libraries created by Facebook to create user interfaces [29]. The biggest advantage of this technology is the easy and fast transition (low entry barriers) from creating websites to writing mobile applications that look and work “natively”, all in the comfort of a JavaScript library. Additionally, most of the written code can be shared between platforms, making it easy to program simultaneously on both Android and iOS.

React Native applications are written using a combination of JavaScript and XML-esque markup, known as JSX. However, the native rendering API for iOS is “written” in Objective-C, and in Java for Android. Therefore, the application created as part of the project is rendered using real mobile user interface (UI) components, not web views, and at the same time looks and functions like any other mobile application. In addition, the above technology also provides JavaScript interfaces for APIs (Application Programming Interfaces) so that applications can access platform features such as the phone’s camera and user location.

React Native allows users to develop applications for iOS and Android platforms using a single set of source code, which helps save time and effort by avoiding the need to write separate codes for each platform. In addition, React Native provides a set of ready-made user interface components that can be easily customized, making interface design more intuitive and efficient. Another advantage of the previously mentioned technology is that the applications created using it tend to run smoothly and responsively, because the JavaScript/TypeScript code is compiled into native code (Java/Kotlin for Android, Swift/Objective-C for iOS), which avoids some delays associated with code interpretation. Another important feature of the technology in question is Hot Reloading, which allows developers to make changes to the code on the fly and see their effects immediately, all in a running application. This helps and speeds up the development and testing process. Nevertheless, the biggest advantage of the above technology is that it has a large and active developer community and a lot of available resources, such as libraries, tools, and tutorials, making it easier to solve problems and gain knowledge.

JavaScript/TypeScript modules in React Native can support logic that does not require direct access to native APIs [41]. In the application, they were used to implement functions such as code formatting, the ability to search, connect, and transfer data using the Bluetooth module implemented in the phone, or connecting to the database via Wi-Fi. The previously mentioned modules are the work of other people or teams, but they have an open-source license, more precisely Apache 2.0.

The application primarily uses the react-native-ble-plx module, which is an open-source library for integrating Bluetooth Low Energy (BLE) functionality into React Native applications. BLE is a wireless communication protocol designed for short-range communication between devices with low power consumption, making it ideal for applications that require wireless communication without quickly draining the device’s battery.

The wireless communication protocol has been optimized for performance and reliability. Communication parameters, power management, and error handling mechanisms have been tuned to ensure smooth and accurate data transfer between devices.

Smooth and efficient navigation between various screens and application components is ensured by the use of the react-native-navigation module, which is also an open-source library. NN aims to provide a native-like navigation experience with animations and performance optimizations, enhancing the user experience and making the app feel more responsive.

Prettier (version 2.8.4) and ESlint (version 8.7.0) are other software modules used in the application project. They are used in development, particularly in JavaScript and related technologies, to ensure code quality, consistency, and adherence to best practices. Although they serve similar purposes, they focus on different aspects of code quality and style. Prettier is a code formatting tool that enforces a consistent and opinionated style across the code base. It automatically analyzes code and rewrites it according to a predefined set of formatting rules. It takes care of details such as indentation, line splits, spacing, and more, eliminating the need for programmers to manually discuss and apply formatting decisions. ESLint is a powerful static code analysis tool that helps identify and fix problems in JavaScript code. It enforces coding standards, detects potential problems, and promotes best practices. Unlike Prettier, which focuses mainly on code formatting, ESLint goes beyond that to identify potential logic errors, bugs, and code smells.

The next software module used is React-i18next, where i18n refers to the word internationalization. It is a library for React applications, including those built with React Native. It helps developers implement multi-language support and localization in their applications, making it easier to create software that can be used by people from different linguistic and cultural backgrounds.

Native-base is the next component of the application, which is an open-source UI component library for building cross-platform mobile applications using React Native. It provides a set of customizable and ready-to-use components that follow Material Design guidelines (for Android) and Human Interface Guidelines (for iOS), allowing developers to create visually appealing and consistent user interfaces for their React Native apps.

To present data and draw charts, the react-native-chart-kit module was used, which is an open-source library that allows for the creation of various types of charts and graphs in React Native applications. It contains a set of customizable chart components that can be used to visualize data in a mobile application. This library is built on top of the react-native-svg library, which enables vector graphics rendering in React Native applications.

NestJS allows building server applications using various protocols. The most common are HTTP-based REST servers. In addition, it provides design architecture, introduces best practices, includes a strong dependency injection mechanism, and uses abstractions, which makes it very flexible in terms of selecting and connecting necessary dependencies, such as databases or libraries. The connection between NestJS and a cloud platform such as Microsoft Azure can be completed in different ways, depending on the specific service or cloud service with which the integration takes place. This project used a model of reading and sending data from/to the server, rather than storing data locally, so as not to take up memory space on the phone or burden the application. In addition, keeping the data in the cloud protects the collected information from being lost and allows charts to be created from a larger period of time than just the last few hours or the last day.

NestJS (version 9.4.2) was used to build the application backend, which is a framework for building efficient, scalable Node.js server-side applications. It uses progressive JavaScript, is built with and fully supports TypeScript (yet still enables developers to code in pure JavaScript), and combines elements of OOP (Object Oriented Programming), FP (Functional Programming), and FRP (Functional Reactive Programming) [42]. Under the hood, NestJS makes use of robust HTTP Server frameworks like Express (the default) and optionally can be configured to use Fastify as well. NestJS provides a level of abstraction above these common Node.js frameworks (Express/Fastify), but also exposes their APIs directly to the developer. This gives developers the freedom to use the myriad of third-party modules available for the underlying platform. One key feature of NestJS is that it includes a modular architecture; in other words, NestJS encourages a modular application structure. It uses modules to organize code into manageable components, making it easier to scale and maintain applications. In addition, the framework is built entirely in TypeScript, a statically typed JavaScript superset. This provides strong typing, better code quality, and improved development tools. NestJS also uses dependency injection to manage the instantiation and injection of components and services. This promotes modularity and testability by enabling the creation of loosely coupled components. The above software uses decorators to define metadata for classes, methods, and properties. They help with tasks such as defining routes, configuring middleware, and more. NestJS uses the popular Express.js framework for handling HTTP requests. This allows for leveraging the benefits of Express while enjoying the additional features provided by NestJS. In addition, the framework supports unit tests, integration tests, and end-to-end tests, and helps ensure application reliability. NestJS also has middleware functions that can be used to process requests and responses globally or locally within specific routes, or validation using libraries such as class-validator. It helps validate incoming data and maintain its integrity. Also worth mentioning is the support for real-time communication using WebSockets via a built-in WebSocket gateway, a command-line interface that simplifies the creation of new modules, controllers, services, and more, and the ability to use NestJS to build microservices using a dedicated module for inter-service communication. NestJS is particularly well-suited for building applications with complex requirements, including APIs, microservices, real-time applications, and more. Its opinionated architecture and comprehensive features make it a powerful choice for backend development in the TypeScript ecosystem.

**Figure 10:**
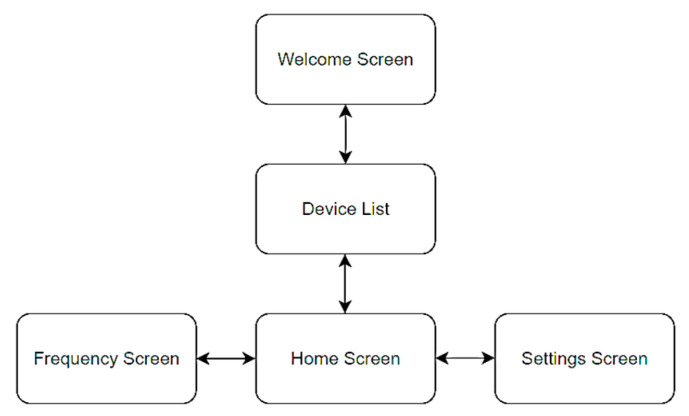
Block diagram of the mobile application.

**Figure 11:**
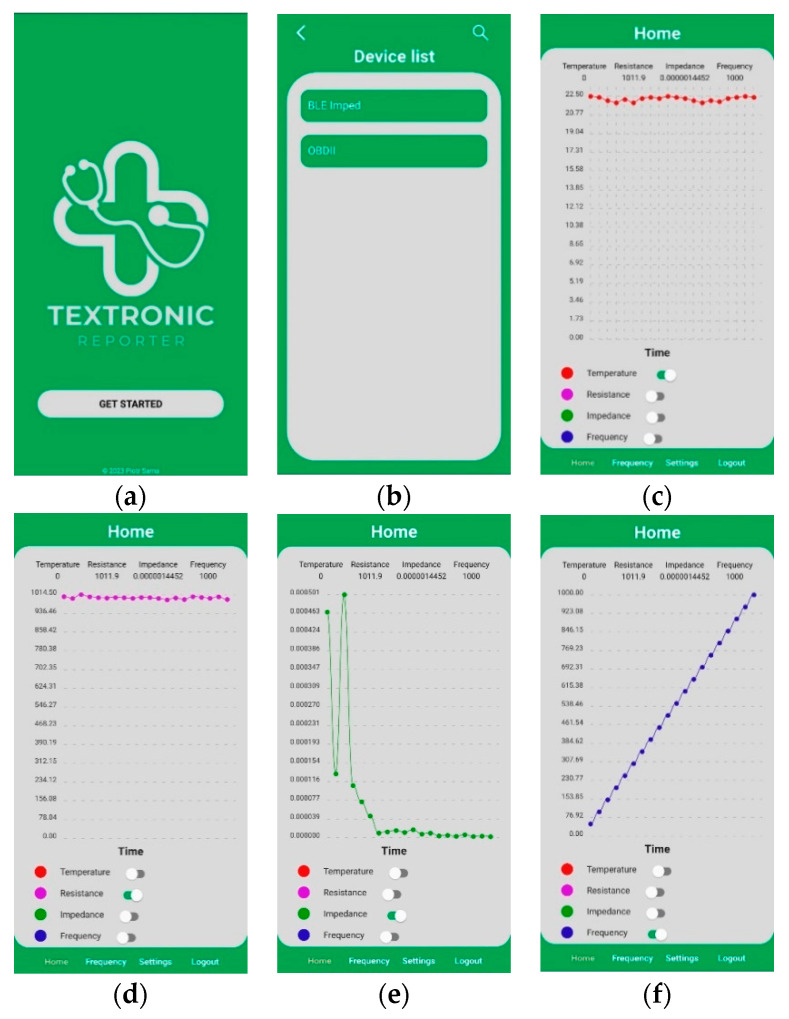
Screenshots of the mobile application. (**a**) Welcome screen (**b**) Device list screen (**c**) Screen with temperature graph (**d**) Screen with resistance graph (**e**) Screen with impedance graph (**f**) Screen with frequency graph. Graphs showing the change of individual parameters over time.

Using the above-mentioned and -described software modules, environments, and frameworks, a mobile application consisting of five screens was created, whose relationship diagram is shown in Figure 10. Meanwhile, the effect of the application is illustrated by copies of the screens shown in Figure 11.

The first interface shown to the user immediately after launching the application is the welcome screen, after which the user is redirected to the list of devices found by Bluetooth. However, for any data to appear, the user must first initialize the search by clicking on the button with the magnifying glass symbol. After selecting the appropriate electronic equipment, the start screen appears, at the bottom of which is a menu, allowing navigation through the other screens, such as displaying the frequency chart or inputs to the settings screen. In addition, there is a logout option in the menu bar that allows the user to return to the list of devices.

After clicking on the Frequency button, an analogous interface to the start screen is shown, with the difference that there is a frequency graph which can only display resistance and impedance values. The last screen is the settings, allowing the user to choose whether the charts should start from zero, whether they should scale to the displayed values, and how much last data should appear on the charts.

It is also worth mentioning the backend of the mobile application, which is responsible for automatically connecting to the measuring system and retrieving data from it, which in subsequent steps are sent to the database and pulled to check whether the process of saving information in the database was successful. It should be borne in mind that the device switches off the Bluetooth module completely after downloading the measurements from it, which forces it to automatically disconnect from the mobile app, and re-initializing the wireless connection must wait until the next measurement is complete.

## 5. Discussion and Conclusions

The results of the work described in the article show the assumptions of the prototype of a collector of data collected from textronic sensors made using physical vacuum deposition technology; however, the proposed solution can be used to measure electrical quantities of other impedance sensors used in wearable electronics. Due to its relatively small size and low energy consumption, the proposed solution can be used in wearable electronics systems for medical applications to control e.g., developing pathogens [2].

To create the prototype of the data collector from textronics structures, nRF52 was used. This module is a cheap platform, but fast ADC measurement and wireless communication proved to be hard to implement due to it being a single core SoC.

React Native was used to create the user interface for the mobile application, as it is a versatile software that uses popular programming languages such as JavaScript, TypeScript, and CSS (Cascading Style Sheets). In addition, it has many ready-made libraries that are frequently updated, allowing users to maintain its compatibility with the latest mobile operating systems. Similarly, the NestJS framework was chosen, with the difference that it already had a ready-made example and documentation allowing immediate communication of the application with the database. For this reason, it was used to transfer data downloaded from the textronic device to a database hosted on Microsoft Azure. The main task of the application is to enable automatic connection to the selected collector and retrieve measurement data, such as resistance, impedance, or temperature, from it. In addition, it allows the display of graphs that show the data selected by the user, and the number of graphs can be easily changed in the settings. The selection of the device to which we automatically connect and retrieve data is performed with the first connection, which initiates data exchange. On the other hand, using logout, this cycle of information enlistment can be interrupted.

## Figures and Tables

**Table 1:** Comparison of indicators for different database management systems [36,37,38,39,40].

Indicators	Azure SQL Database	Microsoft SQL Server	MySQL	PostgreSQL	Oracle
Scalability	Vertical and Horizontal	Vertical and Horizontal	Vertical and Horizontal	Vertical and Horizontal	Vertical and Horizontal
Query performance	High	High	Medium	High	High
Response time	Low	Low	Medium	Low	Low
Integration with data analysis tools	High	High	Medium	High	High
Security	High	High	Medium	High	High
Costs	Subscription-based	Depends on model	Low	Medium	Low
Flexibility and ease of use	High	Medium	High	Medium	Medium

## Data Availability

The data presented in this study are available on request from the corresponding author.
